# Hospital-acquired infections at an oncological intensive care cancer unit: differences between solid and hematological cancer patients

**DOI:** 10.1186/s12879-016-1592-1

**Published:** 2016-06-10

**Authors:** Patricia Cornejo-Juárez, Diana Vilar-Compte, Alejandro García-Horton, Marco López-Velázquez, Silvio Ñamendys-Silva, Patricia Volkow-Fernández

**Affiliations:** Infectious Diseases Department, Instituto Nacional de Cancerología, Av. San Fernando No. 22, Col. Sección XVI, Tlalpan, 14000 México, D.F., Mexico; Department of Critical Care Medicine, Instituto Nacional de Cancerología, Av. San Fernando No. 22, Col. Sección XVI, Tlalpan, 14000 México, D.F., Mexico

**Keywords:** Hospital-acquired infections, Intensive care unit, Multidrug resistance bacteria, Mortality

## Abstract

**Background:**

Cancer patients have a higher risk of severe sepsis in comparison with non-cancer patients, with an increased risk for hospital-acquired infections (HAI), particularly with multidrug resistant bacteria (MDRB). The aim of the study is to describe the frequency and characteristics of HAI and MDRB in critically ill cancer patients.

**Methods:**

We conducted an 18-month prospective study in patients admitted ≥48 h to an ICU at a cancer referral center in Mexico. Patients with hematological malignancies (HM) were compared with solid tumors. Demographic and clinical data were recorded. Mortality was evaluated at 30-days.

**Results:**

There were 351 admissions during the study period, among whom 157 (66 %) met the inclusion criteria of the study as follows: 104 patients with solid tumors and 53 with HM. Sixty-four patients (40.7 %) developed 95 episodes of HAI. HAI rate was 4.6/100 patients-days. MDRB were isolated in 38 patients (24 %), with no differences between both groups. *Escherichia coli* was the main bacteria isolated (*n* = 24), 78 % were extended spectrum beta-lactamases producers. The only risk factor associated with HAI was the presence of mechanical ventilation for more than 5 days (OR 3.12, 95 % CI 1.6 – 6.2, *p* = 0.001). At 30-day follow-up, 61 patients (39 %) have died (38 % with solid tumors and 60 % with HM, *p* < 0.001). No differences were found in mortality at 30-day between patients with HAI (*n* = 25, 39 %) vs. non-HAI (*n* = 36, 38.7 %, *p* = 0.964); neither in those who developed a HAI with MDRB (*n* = 12, 35.3 %) vs. HAI with non-MDRB (*n* = 13, 43.3 %, *p* = 0.51).

**Conclusions:**

Patients with cancer who are admitted to an ICU, have a high risk of HAI, but there were no differences patients with solid or hematologic malignancies.

## Background

Critically ill patients in the Intensive care unit (ICU) are at major risk of Hospital-acquired infections (HAI), related with mechanical ventilation, invasive devices, the use of broad-spectrum antibiotics, and parenteral nutrition, among others [1]. Prompt initiation of appropriate antimicrobial therapy is extremely useful in severely ill patients. After results of cultures are obtained, treatment should be re-evaluated to either de-escalate or escalate the antibiotic prescription. This is associated with optimal costs, decreased incidence of superinfection and minimal development of antimicrobial resistance [2].

Furthermore, cancer patients have a 3- to 5-fold greater risk of severe sepsis in comparison with non-cancer patients, with an increased risk for HAI, particularly with multidrug resistant bacteria (MDRB), which are associated with increased therapeutic failure and high mortality rates [3–8]. Patients with neutropenia or hematological malignancies (HM) appear to be particularly vulnerable to this situation, compared with patients with solid tumors [9].

The aim of this study was to describe the frequency and characteristics of HAI and MDRB in critically ill patients admitted to an ICU at a cancer referral center during an 18-month period, and to compare patients with solid tumors vs. those with HM.

## Methods

The National Cancer Institute (INCan) in Mexico is a 135-bed referral teaching hospital located in Mexico City for adult patients with cancer, with an average of 170,000 medical visits, 7,500 hospital discharges, and 3,500 major surgical procedures per year. The ICU is a six-bed service that serves both surgical and medical patients. All antimicrobial prescriptions are supervised and adjusted on a regular basis by Infectious Diseases specialists.

We conducted an 18-month prospective study of HAI in patients admitted at the ICU from March 2013 to September 2014. The study was approved by the Instituto Nacional de Cancerología Ethics Review Board (“Comité de Etica en Investigación”- INCAN/CI/410/15). Consent was not obtained but patient information was anonymous and de-identified prior to analysis. All patients hospitalized in the ICU for ≥ 48 h were included. The following information was recorded: age, sex, comorbidities, hospitalization and antimicrobials in the previous 3 months, treatment with chemotherapy, radiotherapy or immunotherapy, type of cancer (classified as solid or HM), current status of cancer (complete or partial remission, recent diagnosis, relapsed or progression), stage of disease, reason for ICU admission, time from hospital to ICU admission, severity of illness score using Acute Physiology and Chronic Health Evaluation (APACHE II), organ dysfunctions using Sequential Organ Failure Assessment score (SOFA), length of ICU stay, and ICU mortality rate.

Cultures from blood, urine, surgical site, bronchial secretions, and from any other site with clinical suspicion of infection were performed based on the judgment of the treating physician. Bacteria were cultured using standard microbiological methods. Antimicrobial susceptibility testing was performed by means of the BD Automated Phoenix™ (USA) and the Kirby-Bauer disk diffusion technique in case of resistant strains (Clinical Laboratory Standards Institute, CLSI) [10]. The microorganisms isolated and their susceptibility was recorded. The following MDRB were evaluated: Methicillin-resistant *Staphylococcus aureus* (MRSA); Vancomycin-resistant *Enterococcus faecium* (VRE); Extended-spectrum beta-lactamases (ESBL) *Escherichia coli* and *Klebsiella* spp.; *Pseudomonas aeruginosa*, and *Acinetobacter* spp. resistant to third generation cephalosporins and carbapenems. Other Gram-negative bacteria were considered MDR if they were resistant to fluoroquinolones, third generation cephalosporins, and carbapenems [11].

Infections occurring at more than one site in the same patient were reported as separated infection events, unless the same bacterium was isolated concurrently. HAI were defined using Centers for Disease Control and Prevention criteria (CDC, 2014) [12], and were classified as follows: Central Line-associated Bloodstream Infection (CLABSI); Surgical Site Infection (SSI); Ventilator-associated Pneumonia (VAP); abdominal sepsis, and Catheter-associated Urinary Tract Infections (CA-UTI). Number of ventilator-days, number of central venous catheter (CVC) days, and urinary catheter-days were also recorded.

Mortality and the cause related were evaluated on days 7, 30 and 90, after ICU hospitalization.

### Statistical analysis

The Student *t* test or the Mann–Whitney *U* test were used to compare continuous variables depending on whether they exhibited a normal or non-normal data distribution, respectively, and the Chi-square or the Fisher exact test were used to compare categorical variables. Variables with *P* values <0.5 in the univariate analysis were included in multivariate analysis. A logistic regression analysis was performed for predicting risk for HAI. A cox regression model was performed for survival analysis. Odds ratios (RR) with 95 % Confidence intervals (95 % CI) were calculated. Rates of overall survival were estimated by means of the Kaplan-Meier method and log-rank test. *P* values ≤ 0.05 were considered statistically significant. Device utilization ratios, site-specific cumulative incidence rates per 100 patients, and site-specific incidence densities per 1,000 days at risk or per 1,000 catheter-days were calculated. Data was analyzed using Epi-Info (ver. 7) and STATA (ver. 12) software.

## Results

Three hundred fifty-one patients were hospitalized at the ICU during the study period. One hundred fifty-seven (66 %) patients remained at the ICU for ≥ 48 h and were included in the study as follows: 104 patients with solid tumors (66 %), and 53 with hematological malignancies (34 %). Eighty patients (51 %) were male; mean age was 48 ± 15 years.

Colorectal cancer and liver/biliary tract were the most frequently solid tumors documented. Leukemia (45.3 %) and lymphoma (37 %) were the main HM included. Most of the patients (63.8 %) were in advanced stages of oncological disease stages (III or IV); 27 patients (17.2 %) had metastases.

The most common causes of ICU admission were different between both groups: patients with solid tumors were admitted with hypovolemic shock (26.9 %) and respiratory failure (24 %); while patients with HM had respiratory failure (54.7 %) and septic shock (26.4 %). HM patients had received chemotherapy, radiotherapy or had been hospitalized recently with higher frequency compared with solid tumors. Other clinical and demographic characteristics are shown in Table 1.

**Table 1:** Demographic and clinical characteristics of patients hospitalized at Intensive Care Unit

Characteristic *– n* (%)	Total (*N* = 157)	Solid tumor (*n* = 104)	Hematological malignancies (*n* = 53)	*P*-value
Gender – Masculine	80 (51)	43 (41.3)	37 (69.8)	<0.001
Age (years)^a^	48 ± 15	50.5 ± 15.5	43.9 ± 15.7	0.009
Comorbidities^b^				0.257
Arterial hypertension Diabetes mellitus Smoking Obesity HIV Other	31 (19.7) 29 (18.5) 19 (12.1) 19 (12.1) 2 (1.3) 13 (8.3)	23 (22.1) 23 (22.1) 13 (12.5) 15 (15.4) 1 (1) 8 (7.7)	8 (15.1) 6 (11.3) 6 (11.3) 4 (7.5) 3 (5.7) 5 (9.4)	
Type of tumor^c^				−
NHL HL Acute leukemia Myeloma multiple Myelodysplastic Germ cell tumors Gastrointestinal Cervical Breast Skin and soft tissue Ovarian Head and neck Other tumors	22 (14) 2 (1.3) 22 (14) 4 (2.5) 4 (2.5) 12 (7.6) 23 (14.6) 10 (6.4) 10 (6.4) 10 (6.4) 8 (5.1) 7 (4.5) 20 (12.7)	- - - - 12 (11.5) 23 (22.1) 10 (9.6) 10 (9.6) 10 (9.6) 8 (7.7) 7 (6.7) 20 (19.2)	22 (41.5)^d^ 2 (3.8) 22 (41.5) 4 (7.5) 4 (7.5) - - - - - -	
Stage^e^				
I-II III-IV	38 (36.2) 67 (63.8)	33 (86.8) 5 (13.2)	50 (74.6) 17 (25.4)	0.211
Oncologic status				0.03
Recent diagnosis Progression Relapse Complete remission	96 (61.1) 30 (19.1) 19 (12.1) 12 (7.6)	57 (54.8) 21 (20.2) 14 (13.5) 12 (11.5)	39 (73.6) 9 (17) 5 (9.4) 0	
Chemotherapy in the last 30 days^f^	48 (30.6)	19 (18.3)	29 (54.7)	<0.001
Radiotherapy in the last 6 months	11 (7)	3 (2.9)	8 (15.1)	0.007
Neutropenia^g^	9 (5.7)	4 (3.8)	5 (9.4)	0.02
Pancytopenia	20 (12.7)	1 (1)	19 (35.8)	<0.001
Hospitalization in the last 90 days	45 (28.7)	21 (20.2)	24 (45.3)	0.001
ICU admission				<0.001
Respiratory failure Septic shock Hypovolemic shock Postsurgical Post-CPR Other causes^h^	54 (34.4) 34 (21.7) 29 (18.5) 13 (8.3) 7 (4.4) 20 (12.7)	25 (24) 20 (19.2) 28 (26.9) 13 (12.6) 6 (5.8) 12 (11.5)	29 (54.7) 14 (26.4) 1 (1.9) 0 1 (1.9) 8 (15.1)	
Days from hospitalization to ICU admission^i^	2 (1,8)	0 (1,7)	4 (1,13)	0.02
APACHE-II score^aj^	18.9 ± 6.4	18.4 ± 6.7	19.9 ± 5.6	0.134
SOFA score^ak^	8.6 ± 3.6	8.4 ± 3.9	9.2 ± 3.1	0.197

^a^Mean ± standard deviation

^b^ *HIV* human immunodeficiency virus; other comorbidities: 4 patients with thyroid dysfunction. 1 with chronic kidney failure, 2 with chronic liver failure, 1 with Down syndrome, 1 with ischemic heart disease, 3 with neurologic disease and1 with rheumatologic disease

^c^ *NHL* non-Hodgkin lymphoma; HL = Hodgkin lymphoma; other tumors: lung, bladder, kidney, peripheral nerve, brain

^d^One patient who received hematopoietic stem cell transplantation

^e^Stage was documented in 105 patients: 38 solid and 67 hematologic, percentage was calculated with these numbers. The rest of the patients were not stratified because had a neoplasia unclassifiable

^f^One patient received sorafenib for kidney cancer and one receive bortezomib for multiple myeloma. Three patients received radiotherapy with concomitant chemotherapy

^g^Four patients had anemia plus neutropenia, and five had neutropenia plus thrombocytopenia

^h^Kidney failure, cerebrovascular accident, water-electrolyte imbalance, metabolic acidosis and neurologic impairment

^i^Median (interquartile range)

^j^Acute Physiology and Chronic Health Evaluation

^k^Sequential Organ Failure Assessment

Sixty-four patients (40.7 %) developed 95 episodes of HAI-ICU infection within a median of 7 days from ICU admission (IQR, 4–12 days). VAP was diagnosed in 34 patients (21.7 %), rate of VAP/1000 ventilator-days was 27.6. CA-UTI was the second HAI diagnosed in 22 patients (14 %), rate of CA-UTI/1000 catheter-days was 9.3. When comparing patients with solid tumors vs. those with HM, there were no differences among patients with HAI, VAP, CA-UTI, mechanical ventilation, or ICU length-of-stay. Only CA-UTI/1,000 catheter-days were different, being more frequent in HM patients (*p* = 0.03). The classification of different HAIs and the infections rates are displayed in Table 2.

**Table 2:** Hospital-acquired infections (HAI) at the Intensive Care Unit

Characteristic *–*n (%)	*Total* (*n* = 157)	Solid tumor (*n* = 104)	Hematological malignancies (*n* = 53)	*P*-value
HAI episodes	64 (40.7)	42 (40.4)	22 (41.5)	0.895
MDRB-HAI^a^	38 (24.2)	23 (22.1)	12 (22.6)	0.94
Days of ICU stay^b^	6 (4,11)	6 (4,10)	6 (5, 11)	0.368
Incidence rate per 100 patient-days	4.6	4.6	4.7	0.952
Type of HAI^c^				
VAP^d^ VAP/1,000 ventilator-days CA-UTI CA-UTI/1,000 catheter-days CLABSI CLABSI/1,000 catheter-days SSI^e^ Abdominal sepsis	34 (21.7) 27.6 22 (14) 9.3 4 (8.9) 1 13 (8.3) 3 (1.9)	22 (21.2) 27.3 13 (12.5) 7.4 2 (1.9) 0.8 13 (12.5) 2 (1.9)	12 (22.6) 28.2 9 (17) 14.9 2 (3.8) 1.3 1 (1.9) 1 (1.9)	0.83 0.971 0.444 0.03 0.603 0.259 0.03 1

^a^ *MDRB* multidrug-resistant bacteria (ESBL- *Escherichia coli*; Multidrug-resistant-extensively drug-resistant (MDR/XDR)- *Pseudomonas aeruginosa*; MDR- *Acinetobacter baumannii*; Methicillin-resistant *Staphylococcus aureus*, Vancomycin-resistant enterococcus)

^b^Median Interquartile (IQ) range

^c^ *VAP* ventilator associated pneumonia, *CA-UTI* catheter related urinary tract infection, *CLABSI* central line associated bloodstream infection, *SSI* surgical site infection

^d^131 patients required mechanical ventilation

^e^Surgery was performed in 72 patients

**Fig. 1:**
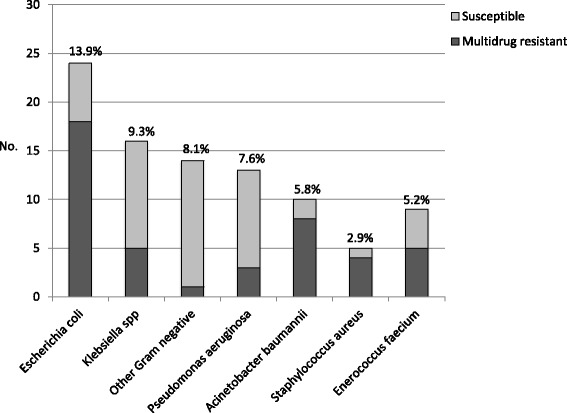
Main bacteria isolated in patients with Hospital-Acquired Infection at the Intensive Care Unit (HAI-ICU), divided into susceptible and multidrug-resistant bacteria (MDRB). The percentage was obtained from the total number of microbiological isolates (*n* = 172)

During the study period, 910 cultures were taken; 172 (18.9 %) microorganisms were identified, including 102 Gram negative (59.3 %), 46 Gram positive (26.7 %), and 24 yeasts (14 %). MDRB were identified in 35 patients (24.2 %), with no differences between both groups (*p* = 0.94). *Escherichia coli* was the most frequent bacterium isolated (78 % were ESBL producers). Other bacteria are shown in Fig. 1.

Regarding fungi, there were seven candidemia episodes (three due to *Candida glabrata,* two due to *C. tropicalis*, one due to *C. parapsilosis*, and one due to *C. albicans*). Three candidemia episodes were associated with severe neutropenia, two with CVC infection, and two with parenteral nutrition. Six of these patients died (86 %), in a mean time of eight days since *Candida* growth. *Aspergillus* spp. was identified in broncho-alveolar samples, three patients (two *A. fumigatus* and one *A. flavus*), all with hematologic malignancies, severe neutropenia and lung nodules in scan tomography. Two patients with *Aspergillus* died.

Concomitant infections were documented in 19 patients; two different bacteria were documented in 15 patients (three with abdominal sepsis, eight with pneumonia and four with SSI), one patient had influenza virus with a bacterial co-infection, one patient with CLABSI had a Candida plus a bacteria, and two patients with SSI had two different bacteria plus Candida.

In the logistic regression model, over five days of mechanical ventilation was the only statistically significant factor for HAI (OR 3.12, 95 % CI 1.6 – 6.2, *p* = 0.001).

30-days overall mortality was 61 patients (39 %): 29 (28 %) with solid tumors and 32 (60.3 %) with HM (*p* < 0.001).

**Fig. 2:**
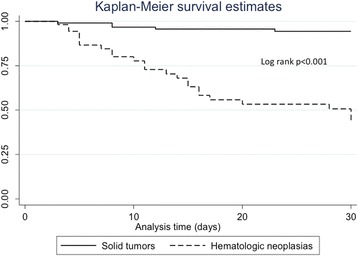
Kaplan-Meier survival curve. 30-day mortality of patients with solid tumors vs. hematologic malignancies

Sixty-one patients died at 30-day follow-up, no differences were found between patients with HAI (*n* = 25, 39 %) vs. non-HAI (*n* = 36, 38.7 %, 0.964); neither at 90-day follow-up (*n* = 27, 42.2 % vs. *n* = 52, 55.9 %, respectively, *p* = 0.09). No differences were found in mortality at 30-day between patients who developed a HAI with MDRB (*n* = 12, 35.3 %) vs. HAI with non-MDRB (*n* = 13, 43.3 %, *p* = 0.51) Fig. 2.

## Discussion

In oncological patients, the interaction of multiple factors related to immune supression, skin or mucosal disruption, extensive antibiotic use, complex surgical procedures and the presence of invasive devices, augment the risk of HAIs, leading to an increase in the length of hospital stay, morbidity and mortality [13].

As a result, the number of patients admitted to the ICU, either for cancer-related complications or for treatment-associated side effects is steadily increasing [9].

Although we did not specify the chemotherapy regimens, it is important to consider that hematologic patients, particularly with acute leukaemias, aggressive regimens amplify disease-induced immunosupression and result in severe and long lasting neutropenias which could favour the development of severe infections. This was not confirmed in this study, most likely because of the low number of patients with this disease.

Some studies describe as the main reasons to the ICU admission, septic shock, respiratory failure and hypovolemic shock secondary to extensive surgical resection in patients with solid tumors. In this study we found differences for ICU admission in both groups: in patients with HM the most frequent cause was respiratory failure (54.7 %), it was related to several causes such as pneumonia, acute respiratory distress syndrome, diffuse alveolar hemorrhage and pulmonary embolisms. However, in patients with solid tumors the main reason for ICU admission was hypovolemic shock secondary to schedule or emergency surgical resection (29.9 %). This percentage was higher than the one reported in other study performed in the same ICU (31.6 % for septic and hypovolemic shock) [14]. Reports from other parts of the world not even include hypovolemic shock within the first causes of admission to ICU [15–17]. One possible explanation for the high percentage we found, is that patients admitted to our hospital have advanced cancer stages, with tumor adhesion to adjacent organs, therefore require extensive surgeries, with multiple organ resection, which increases bleeds and surgical morbidity.

HAI prevalence varies among hospitals and among countries. There has been reported an increase in patients with cancer during the last decade [18]. Some studies have reported a wide variation rate between 5.3 and 56.1 % [19]. Our HAI rate was 40.7 %. The most frequent infection was VAP (26.7 %, 28.4/1,000 ventilator-days). The VAP rate was similar to the ones described in other ICUs in developing countries (24.1/1,000 ventilator-days) [20]. More than five days of mechanical ventilation was the only risk factor associated to HAI in multivariate analysis (OR 3.12, CI 95 % 1.6 – 6.2, *p* = 0.001), as has been reported previously [21].

Surgical site infection (SSI) was the second HAI documented: 72 surgeries were performed during the study period; the infection rate was 16.7 (the SSI infection rate reported during 2013 in our institution was 9.9). It is relevant to highlight that 28 % of the surgeries were emergency procedures, and the majority of these comprised complicated and extensive interventions.

In various reports, CA-UTIs are described as the most common nosocomial infection (around 40 %) [22]. These infections do not cause severe mortality or morbidity, but can progress into severe infections such as secondary bacteremia or septic shock, in addition to significantly increasing the hospital costs [23]. We found CA-UTI as the third HAI in the ICU (13.4 %, 8.9 infections/1,000 catheter-days), as the same as reported by other authors [20]. Median of indwelling urinary catheter stay were 16 days in patients with CA-UTI compared with 10 days in non-CA-UTI patients.

The CLABSI rate was 2.5 % (1 infection/1,000 catheter-days). In some U.S. studies, which report prevalence, the range varies from 1.8 to 7.6/1,000 catheter-days [24]. In one research conducted in eight developing countries, the rate varied between 4.2 and 14.4/1,000 catheter-days [17]. The low rate found in this series is attributed to strict standardized protocol of care provided by an intravenous therapy team nurses working for over two decades [25, 26].

*E. coli* was the most frequent pathogen isolated, 78 % were ESBL producers. This percentage is considerably higher than reported elsewhere in our country (range between 33 and 51 %) [27–29], but it is important to note that these studies included patients hospitalized in different wards, not only from ICU where the prevalence of MDRB is usually higher.

In recent years, widespread MDRB in hospitals had complicated the medical care and infection prevention measures. MDRB pathogens are associated with worse outcomes related with inferior therapeutic options and delay in initiation of appropriate therapy, and probably with increased virulence of these strains [30]. In this study we found 24.2 % of MDRB, which is lower when compared to a retrospective study performed from 2007 to 2011 in the same ICU, when the HAI rate was 39.7 % [31]. In another Mexican study with patients admitted in ICU, the incidence of MDRB was 64.5 % [32].

In this report, overall 30-day mortality was 39 % with ranges between 28 % in patients with solid tumors until 60.4 % in HM group. The poor prognosis of patients with HM, especially those who received hematopoietic stem cell transplantation (HSCT), has been well documented [17]. We did not have HSCT patients because they are treated exclusively in the transplant unit. We found a difference in mortality related with the cause of admission to ICU: 60 % in patients admitted with respiratory failure, 29.4 % in patients with septic shock and 10.3 % in scheduled surgical patients, but this could be related with the oncology disease, because HM patients had also more frequently respiratory failure. In another study, that included oncology patients admitted at the ICU, the overall hospital mortality was 30 %, (range, 10 % to 70 %) [9, 17].

Early management of organ dysfunction is crucial in the treatment of critically ill patients; late ICU admission has been associated with greater mortality [9]. We found a significant delay in ICU admission in patients with HM (median 2 days compared solid tumors median 0 days), which could be also related with higher mortality in these patients.

Infection control measures are strict in our ICU: at admission, two blood cultures, urine, respiratory secretions and other cultures are required; broad-spectrum antibiotics are initiated as soon as patient arrival. An antimicrobial re-evaluation is made in the first 48–72 h when cultures results are available and according to clinical evolution to either de-escalate or escalate the antibiotic prescription; antibiotics schemes are discussed and reviewed periodically with all staff (intensive care, infectious disease and medical or surgical oncologists physicians); the duration of antimicrobials treatment are shortened as much as possible; surveillance hand hygiene program, and follow antibiotic prophylaxis guides [2].

Some limitations of this study are that the results correspond to a single-center and did not obtain data related to the long-term survival. However, we believe these results provide insight into the prevalence of HAI, particularly MDRB at a cancer referral center, and can be extrapolate results to other hospitals with similar characteristics.

## Conclusions

Our study indicates that the prevalence of HAI is similar than other reports performed in cancer patients. There were no differences between patients with HAI vs. non-HAI, neither in those who had a MDRB isolated. Adherence to the guidelines for the prevention and control of infections are the most useful tool to decrease HAI infections and improve outcomes in susceptible patients.

## Abbreviations

APACHE II, Acute Physiology and Chronic Health Evaluation II; CA-UTI, Catheter-associated urinary tract infections; CLABSI, Central line-associated bloodstream infection; CVC, Central venous catheter; ESBL, Extended-spectrum beta-lactamases; HAI, Hospital-acquired infections; HCT, Hematological stem cell transplantation; ICU, Intensive care unit; MDRB, Multidrug resistance bacteria; SOFA, Sequential Organ Failure Assessment; SSI, Surgical site infection; VAP, Ventilator associated pneumonia
